# Septic Olecranon Bursitis With Osteomyelitis Attributed to Cutibacterium acnes: Case Report and Literature Overview of the Dilemma of Potential Contaminants and False-Positives

**DOI:** 10.7759/cureus.34563

**Published:** 2023-02-02

**Authors:** John G Skedros, Ethan D Finlinson, Meredith G Luczak, John T Cronin

**Affiliations:** 1 Shoulder and Elbow, Utah Orthopaedic Specialists, Salt Lake City, USA; 2 Department of Orthopaedics, University of Utah, Salt Lake City, USA

**Keywords:** p. acnes, propionibacterium acnes, contaminants, false-positive, osteomyelitis, c. acnes, cutibacterium acnes, olecranon bursitis

## Abstract

We report an unusual case of acute septic olecranon bursitis, with probable olecranon osteomyelitis, where the only organism isolated in culture was initially considered a contaminant, *Cutibacterium acnes*. However, we ultimately considered it the likely causal organism when treatment for most of the other more likely organisms failed. This typically indolent organism is prevalent in pilosebaceous glands, which are scarce in the posterior elbow region. This case illustrates the often challenging empirical management of a musculoskeletal infection when the only organism isolated might be a contaminant, but successful eradication requires continued treatment as if it is the causal organism. The patient is a Caucasian 53-year-old male who presented to our clinic with a second episode of septic bursitis at the same location. Four years prior, he had septic olecranon bursitis from methicillin-sensitive *Staphylococcus aureus* that was treated uneventfully with one surgical debridement and a one-week course of antibiotics. In the current episode reported here, he sustained a minor abrasion. Cultures were obtained five separate times because of no growth and difficulty eradicating the infection. One culture grew *C. acnes* on day 21 of incubation; this long duration has been reported. The first several weeks of antibiotic treatment failed to eradicate the infection, which we ultimately attributed to inadequate treatment of *C. acnes* osteomyelitis. Although *C. acnes* has a well-known propensity for false-positive cultures as typically reported in post-operative shoulder infections, treatment for our patient’s olecranon bursitis/osteomyelitis was successful only after several surgical debridements and a prolonged course of intravenous and oral antibiotics that targeted it as the presumptive causal organism. However, it was possible that *C. acnes* was a contaminant/superinfection, and another organism was the culprit, such as a *Streptococcus* or *Mycobacterium* species that was eradicated by the treatment regime targeted for *C. acnes*.

## Introduction

Orthopaedic surgeons and other healthcare providers who focus on musculoskeletal conditions are periodically faced with situations where there is clearly an infection, but the cultures fail to grow an organism or only show rare growth of an unlikely causal organism [1,2]. There are several explanations for this, including the administration of pre-culture antibiotics and inadequate culture media/conditions, especially for anaerobic biofilm-producing microorganisms [1,3,4]. In our shoulder and elbow reconstruction orthopaedic surgery practice we have become especially aware of the problem of negative or rare-growth cultures in deep infections of the shoulder region, especially after rotator cuff repairs and prosthetic arthroplasties [5-9]. Nearly 20 years ago when there was rare growth of an organism, such as a coagulase-negative *Staphylococcus* sp. or *Cutibacterium acnes* (formerly *Propionibacterium acnes* [10]) from deep tissue or fluid cultures obtained from post-operative shoulder infections it was often deemed a contaminant [5]. Empirical antibiotic treatment then ensued for a presumptive organism that failed to grow in culture. It was eventually realized that these more indolent organisms were the cause of some of these less-clear cases. Back then, surgeons needed to request that microbiology laboratories keep tissue cultures for 14 days so that these indolent organisms could be detected, especially for *C. acnes*. It is now routine protocol for microbiology laboratories to hold tissue cultures from bone and joint infections for 14 days, which is necessary to detect *C. acnes* [11]. However, sometimes up to 21 days is needed for this organism [12].

In the present case we were faced with a situation where, similar to confusing cases of post-operative shoulder infections, there was only the rare growth of an anaerobic/indolent biofilm-forming organism (*C. acnes*). *C. acnes* seemed to be a very unlikely cause of our patient’s acute septic olecranon bursitis with osteomyelitis because of the paucity of pilosebaceous glands, where *C. acnes* reside, in that region. There is only one reported case where *C. acnes* was considered a probable cause of septic olecranon bursitis, but that case also grew *Staphylococcus epidermidis* [13]. Hence, one of those organisms may have been a contaminant or superinfection. We report the present case of septic olecranon bursitis for three main reasons: (1) the rare growth of only *C. acnes* in culture, which was initially considered a contaminant but was eventually viewed as a likely cause of the infection, (2) to illustrate challenges in the treatment of a deep infection when it is unclear if the cultured organism is the cause of the infection or a contaminant/superinfection, and (3) show that successful treatment of a putative causal organism like *C. acnes* can also successfully treat other possible causal organisms that failed to grow in culture.

## Case presentation

**Figure 1:**
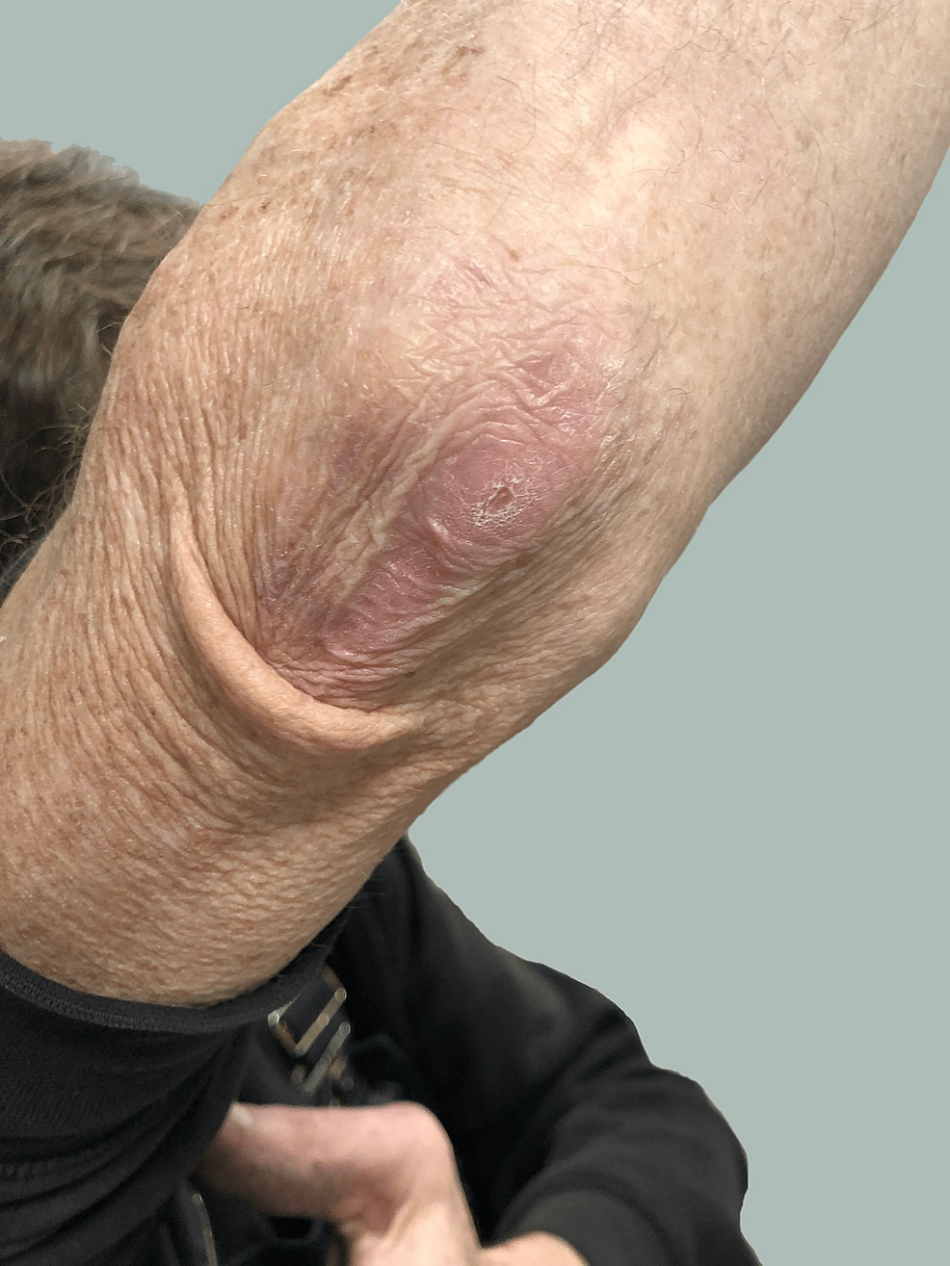
Patient's Posterior Elbow at 10 Months After Completing Antibiotic Treatment At the proximal aspect of the incision there is an asymptomatic adhesion (causing the obvious skin crease) stemming from the debridement surgery that was done four years prior. This image was taken in 20˚ of elbow flexion.

The patient is a 53-year-old right-hand-dominant male (height: 183 cm; weight: 84 kg; BMI: 25.13) who presented to our clinic with a chief complaint of pain and swelling at the posterior aspect of his right elbow. He did not have diabetes, denied illicit drug use, and did not have prior corticosteroid injections into his olecranon bursa. He smoked one pack of cigarettes and drank six or more beers each day. During his job installing fire sprinkler systems in commercial buildings, he would sustain abrasions on his elbows, forearms, and hands. Four years prior, an abrasion of his right elbow led to septic olecranon bursitis with methicillin-sensitive *Staphylococcus aureus*. This resolved after one surgical debridement and a one-week course of intravenous (IV) cephazolin and oral cephalexin. However, as a result of that debridement, he developed a painless scar adhesion between the skin and deeper tissues (Figure 1).

Prior to coming to our clinic for his second episode of septic olecranon bursitis, the patient’s primary care physician (PCP) empirically prescribed oral cephalexin 500mg four times a day and oral sulfamethoxazole/trimethoprim double strength twice a day; this treatment regime covered a broad spectrum of *Staphylococcus* and *Streptococcus* species that are common causes of septic olecranon bursitis [14]. No aspiration was done because there was no effusion. One week later, he was seen in our hospital emergency department (ED) (October 1, 2017) with worsening redness and progressive swelling of the posterior elbow region. Bloody fluid aspirated from the bursa was cultured. Blood tests showed normal values (basic metabolic analysis, white blood cell count [WBC], erythrocyte sedimentation rate [ESR], and C-reactive protein [CRP]). An additional 10-day course of the same oral antibiotics was prescribed. Five days later, his PCP aspirated bloody fluid from the olecranon bursa because of no growth from the ED culture. The patient continued the same antibiotic regimen. The PCP’s culture eventually grew *C. acnes* on day 21 (Table 1).

**Table 1:** Culture Dates, Incubation Times, Antibiotics, and Results * C = cephalexin; S/T = sulphamethoxazole/trimethoprim

Date of Culture	Fluid or Tissue	Days Held	Antibiotics*	Growth
Dec. 1, 2017	Fluid	14	C and S/T	None
Dec. 6, 2017	Fluid	21	C and S/T	C. acnes
Dec. 7, 2017	Tissue and Fluid	21	C and S/T	None
Dec. 18, 2017	Tissue and Fluid	21	C and S/T	None
Dec. 26, 2017	Tissue	21	vancomycin, levofloxacin, and rifampin	None

At his first visit to our clinic (PCP culture was on day three), his olecranon bursa was moderately swollen and erythematous, but was not draining. The first irrigation and debridement procedure was then done using sterile technique. Through two 1.5-centimeter incisions, one at the proximal and one at the distal aspect of the bursa, 10 milliliters of seropurulent/phlegmonous material was drained. The inflamed bursa lining was scraped with a curette and the tissue was sent for aerobic and anaerobic bacterial, acid-fast bacilli (AFB/*mycobacteria*), and fungal cultures using the Isolator and BACTEC systems (Wampole Laboratories, Cranbury, NJ, USA; Becton Dickinson Diagnostic Instruments Systems, Sparks, MD, USA; [https://www.testmenu.com/Intermountain/TestDirectory/SiteFile?fileName=sidebar%5CWhichSwabForCulture-160622.pdf], accessed January 26, 2023). Irrigation was then done with 3% hydrogen peroxide, followed by saline, and then packing with an iodine-containing gauze strip. The current oral antibiotics were continued, reflecting our opinion that the missed aspect of his treatment was a debridement [15]. During the next 10 days, four debridements were done in the clinic and each included a sterile prep, gentle curettage of the bursa lining, and insertion of new sterile gauze packing strip. Although progressive improvement was seen, mild serous discharge persisted.

**Figure 2:**
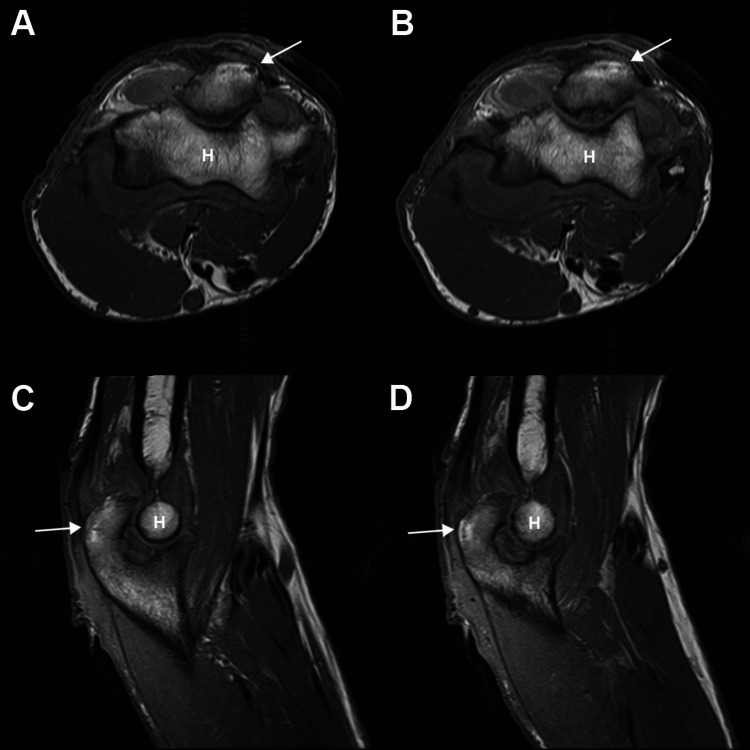
MR Images of Patient's Elbow Magnetic resonance (MR) T1-weighted images of our patient’s elbow in axial (A and B) and sagittal (C and D) views. Arrows point to increased signal in the olecranon, suggestive of early osteomyelitis. “H” indicates the condylar region of the humerus. Arthritis of the elbow joint, primarily of the olecranon articular surface, is also seen in the sagittal views.

Because of continuing serous discharge and lack of growth from three prior cultures (ED, PCP, and ours) we again cultured the bursa lining (Table 1). MRI scanning revealed mild edema of the adjacent bone and marrow, suggesting early osteomyelitis (Figure 2). Consequently, daily oral levofloxacin 750mg and IV vancomycin were started (October 22, 2017) for broader coverage, as recommended by an infectious disease consultant [16]. Aspiration of the elbow joint yielded only a few drops of normal-appearing synovial fluid. After four days of this oral/IV antibiotic treatment, we performed a formal surgical irrigation and debridement with closure over a suction drain. Notably, none of the five debridements were deeper than the periosteum.

All cultures obtained during the patient’s care were sent to the same laboratory. One culture was held for 14 days, all others were held for 21 days at the request of his surgeon (JGS) [11,17,18]. Our lab used fortified thioglycolate broth culture in anaerobic conditions. Because of the recalcitrant nature of the infection and growth of only *C. acnes* in culture, antibiotics were switched to IV ceftriaxone 2 grams daily and oral rifampin 300mg twice a day [19]. Despite improvement over the next three weeks, there was a 2mm region of the incision that had trace erythema with several drops of serous drainage per day. Consequently, our infectious disease consultant stopped oral rifampin and­­­ re-started IV vancomycin for an additional three weeks (ceftriaxone was also continued) for presumed osteomyelitis.

All antibiotics were stopped on December 31, 2017, and the infection was considered eradicated. However, two weeks later mild erythema and slight serous discharge recurred. Oral amoxicillin 500mg every six hours was then prescribed, which he took for three months [20,21]. We speculated that this extended course of oral antibiotics following IV antibiotics was the consequence of several factors, including (1) the ability of *C. acnes* or another anaerobic bacteria species to form a biofilm, (2) our patient’s immunocompromised state and poor healing capacity as a result of his alcoholism and smoking, (3) prior case of septic bursitis leaving subcutaneous scar tissue, and (4) bone involvement [22,23]. However, as discussed below, we also realized that other biofilm-forming organisms that failed to grow in culture might have been the causal organism and *C. acnes* was a contaminant/superinfection. No recurrence had occurred by his final follow-up 4.5 years later.

## Discussion

*C. acnes*, commonly known for its role in acne, is an anaerobic Gram-positive bacterium skin commensal, especially in hair follicles (pilosebaceous glands) and the dermis [12,24]. In contrast to the posterior elbow where pilosebaceous glands are scarce, *C. acnes* is abundant in the shoulder region [25]. Consequently, there is ample literature with respect to the treatment of *C. acnes* infections in the settings of shoulder corticosteroid injections, rotator cuff repairs, and shoulder arthroplasties [5,18,26,27]. In these cases, penicillins (penicillin G and amoxicillin) and cephalosporins (cephalothin and ceftriaxone) have been shown to be effective in killing *C. acnes* [28]. Much can also be learned from shoulder surgery literature about *C. acnes* as a contaminant or false-positive organism in deep infections. For example, in a study on the incidence of *C. acnes* in open shoulder surgeries in cases that were not infected, Mook et al. [29] reported that 20.5% of surgeries (24 of 117) yielded at least one positive culture specimen. Notably, *C. acnes* represented 83% (39 of 47) of all positive cultures. They hypothesized that the unusually high prevalence of *C. acnes* infections reported in the shoulder surgery literature is likely due to cultures that are false-positive for infection. False-positive cultures might result from contamination during specimen collection, such as inadvertent inoculation of *C. acnes* from the skin, transport, incubation, or laboratory error [29]. From the perspective of these data, we initially assumed that *C. acnes* was a skin contaminant and that the organism that was the true cause of our patient’s infection was suppressed by the ongoing antibiotic treatment.

Because of our patient’s recalcitrant infection, we eventually viewed *C. acnes* as the likely cause, which was supported by its well-known propensity to form a biofilm in addition to being anaerobic. Few other organisms reported in cases of septic olecranon bursitis (Table 2; [13,23,30-51]) seemed more likely. It is possible that our patient’s infection was caused by a *Streptococcus* species, which are a relatively more common cause septic olecranon bursitis (Table 2), and *C. acnes* was a superinfection that was introduced during the multiple debridements [52,53]. A serum antistreptolysin O (ASO) titer would have helped detect Streptococcal species [54] but, unfortunately, this was not obtained from our patient. Additionally, despite the negative culture results, it is possible that other slow-growing atypical microbes, such as a *Mycobacterium* species, may have been the causative organism especially when considering that our patient was in an immunocompromised state from his alcoholism [31,34,51,55,56]. Many *Streptococcus* and *Mycobacterium* species also form biofilms and most of these species are also anaerobic [57-59]. The presence of such organisms may have been masked by the partial treatment that he received with levofloxacin and rifampin, thus explaining some response to these microbes followed by remission due to prolonged IV antibiotics. These possibilities further highlight the dilemma that clinicians may face when cultures show sole growth of an unlikely organism, and improvement in the infection is only seen when empirical treatment is continued for one or more putative causal organisms.

**Table 2:** Organisms Cultured from Bursitis Aspirates† † This table resembles the original published by Reilly and Kamineni [50], “Olecranon Bursitis”, pg. 162, copyright Elsevier; reproduced with permission. The original table has incorrectly numbered references, with most being off by one number. For example, if the actual reference was supposed to be no. 7, they indicated it as no. 8. In some cases, they list organisms that were not mentioned by the referenced study. For example, Weinstein et al. [47] or Wasserman et al. [48] (unclear which was intended) was listed as reporting Group A *Streptococcus*, but neither did. * Reilly and Kamineni did not distinguish between olecranon and other bursae. Consequently, it is also not clear which bursa is being referred to (e.g., olecranon or pre-patellar bursa) in some cases (indicated above with an asterisk). Although we attempted to focus on organisms cultured from olecranon bursae in our expansion of their Table, this was not always clear (like *C. acnes* in Pien et al. [34]). Note that we show *Streptococcus pyogenes* separate from Group A because that species was exactly specified in the three studies listed. ‡ This recent report describes *Staphylococcus epidermidis* and *Propionibacterium acnes* (*C. acnes*) as isolates from a case of olecranon bursitis/osteomyelitis [13].

Correction and Expansion of Table 1 in Reilly and Kamineni [50]	
Organism	Reference
Gram-positive cocci	
*Staphylococcus aureus*	Most commonly found in most studies
*Staphylococcus epidermidis*	[13]^‡^, [23]*, [30], [31]*, [32], [33]*, [34]*, [35], [36], [37]*
Group A *Streptococcus*	[23]*, [30], [31]*, [32], [33]*, [35], [37]*, [38], [39], [40]*, [44]
Group B *Streptococcus *(includes* S. agalactiae*)	[30], [31]*, [37]*
Group C *Streptococcus*	[35]
Group G *Streptococcus*	[36], [38], [40]*
*Streptococcus pyogenes*	[40]*, [41]*, [42]
*Strept. pneumoniae* (& other α-hemolytic)	[40]*, [43]
*Enterococcus faecalis*	[41]*
Gram-positive bacilli	
*Clostridium perfringens*	[44]
*Bacillus subtilis*	[23]*
Gram-negative bacilli	
*Acinetobacter anitratus*	[34]*
*Klebsiella oxytoca*	[41]*
*Escherichia coli*	[31]*, [34]*
*Enterobacter cloacae*	[23]*, [34]*, [44]
*Serratia marcescens*	[45]
Gram-negative coccobacilli	
*Pseudomonas aeruginosa*	[31]*
*Pseudomonas fluorescens*	[23]*
*Haemophius influenzae* (type b)	[44], [46]
Anaerobes	
*Brucella abortus*	[31]*
*Cutibacterium acnes*	[13]^‡^, [34]*
Mycobacteria	
*Mycobacterium kansasii*	[49]
*Mycobacterium tuberculosis*	[31]*
*Mycobacterium marinum*	[34]*
Mycobacterium asiaticum	[51]

For clinicians who face similar circumstances in a patient with septic olecranon bursitis where *C. acnes* is the only organism grown in culture, it is useful to consider the probability that it is the causal organism in the perspective of data showing that it is difficult to grow this organism in culture. The dilemma of rare-growth or no-growth cultures is now recognized as common in post-operative musculoskeletal infections, especially of artificial joints [1]. The incidence of negative cultures in this context ranges from 0 to 47% [1,60-65], with the highest percentage from non-arthroplasty shoulder surgeries [1]. A previous study focused specifically on olecranon and prepatellar septic bursitis, reported a culture-negative infection rate of nearly 30% [2]. In addition to being anaerobic, *C. acnes* grows slowly in culture for several reasons including the presence of a polymicrobial infection, culture media used, and other factors [11,17,18]. Because of this slow growth, *C. acnes* has potentially higher false-positive rates for long-hold specimens [6,29,66]. However, Bossard et al. [11] showed that reducing the culture time of *C. acnes* from 14 days to seven days increased the false-negative diagnostic rate by 21.4%. For the optimal recovery of *C. acnes* from surgical tissues, Hsu et al. [67] reviewed the literature and concluded that specimens should be cultured on multiple media, including aerobic, anaerobic, and broth, and should be observed for at least 17 days. Some studies suggest that cultures for *C. acnes* should be held up to 21 days [12], as was done in four of five cultures obtained in our case. In 51 non-infected patients that had cultures obtained at the time of shoulder arthroscopy, Chuang et al. [12] reported that they would have missed 34% of their positive cultures for *C. acnes* if the cultures were held for only 14 days. Some investigators now advise that a *C. acnes* infection should be diagnosed only when there are multiple positive cultures [17]. However, this recommendation is typically for establishing the presence of the causal organism in deep post-operative infections where a prosthesis or other orthopaedic hardware is present. But when there is no prosthesis/hardware and multiple cultures are not taken at the same time (as in our case), the rare growth of a prevalent commensal organism like *C. acnes* poses a dilemma.

We did not pause our patient’s antibiotic treatment so that cultures could be grown without the suppression of antibiotics. Current methods that can help to distinguish between contamination and true infection include obtaining multiple cultures at the same time, histopathological analysis, distinguishing single bacterial cells from biofilm-related infections by immunofluorescence microscopy, investigation of the ability of *C. acnes* isolates to form a biofilm, and other emerging methodologies [68,69]. Osteomyelitis in the adjacent bone has been reported as a complication of septic olecranon and pre-patellar bursitis [13,30,31,44,70]. In our patient, the *C. acnes* and/or another organism that failed to grow in culture might have been introduced by a deep puncture/abrasion. Microscopic spread of the organisms might be caused by cell division and/or the intracellular invasion of marrow mesenchymal stem cells, which is a known behavior of *C. acnes* [71]. This type of spread may have been more likely in our patient since the skin over his olecranon was adherent to the underlying tissues, suggesting a more direct path from skin to bone (Figure 1). Hence, the bone itself may have become the reservoir of biofilm-encased *C. acnes* that caused our patient’s recalcitrant septic olecranon bursitis. However, *C. acnes* osteomyelitis is rare [72,73]. Therefore, in the treatment of our patient it would seem prudent to debride and culture the bone as well. However, given that our patient was a manual laborer who was concerned about returning to work quickly and the possibility of further surgery for muscle flap coverage of the debrided bone [74,75], we opted to avoid debridement of the olecranon. After our patient successfully cleared his infection, it became clear to us that there have been reports of septic olecranon bursitis with osteomyelitis that utilized bone debridement without subsequent plastic surgery intervention for wound coverage (e.g., no muscle flap was required) [76,77]. Therefore, physicians who are faced with similar circumstances should consider more aggressive debridement than what was done in this present case.

Because *C. acnes* was the only organism grown in culture, the possibility that it was the causal organism of our patient’s olecranon bursitis/osteomyelitis warrants additional critical consideration in the context of other possible causal organisms. There are *C. acnes* strains that are invasive and others that are not; the latter are usually studied [68,78]. *C. acnes* is also known to cause osteomyelitis as a complication of implanted orthopaedic hardware [20,71,79-81]. Notably, some Mycobacterial and Streptococcal strains can also be locally invasive and cause osteomyelitis [55,82-84]. Though the prevalence of *C. acnes* as the cause of osteomyelitis is not known, results of a study by Asseray et al. [80] further illustrate the rarity of our patient’s case. These investigators conducted a retrospective study with the aim of developing a diagnostic strategy to distinguish *C. acnes* infections from other bacterial contamination. Their cohort included all patients hospitalized between 2000 and 2004 in an orthopaedic surgery unit who had a deep sample positive for *C. acnes* from the pelvis (15% of cases), spine (40%), lower limb (12%), shoulder (22%), and other upper limb (11%) (the elbow was not specified). Although 65 patients had cultures positive for *C. acnes*, only 52 (80%) were considered to have a true infection. Of those 52 patients and unlike our patient, 48 had an implanted orthopaedic device, likely reflecting a thicker or otherwise modified *C. acnes* biofilm in association with the metal [71,85]. We could not locate another case where septic olecranon bursitis with osteomyelitis was possibly caused only by *C. acnes*. Septic bursitis due to *C. acnes* has been reported in other bursae, such as the iliopectineal bursa [86]. Prior reports of septic olecranon bursitis attributed to *C. acnes* are almost always inaccurately or imprecisely referenced (Table 2). For example, Pien et al. [34] reported a case of *C. acnes* bursitis, but they did not report and could not recall if their patient had olecranon or pre-patellar bursitis (personal communication, Dr. Francis Pien). Furthermore, they did not provide details of the patient’s characteristics, potential cause, or treatment rendered. The only other case that reported *C. acnes* olecranon bursitis was polymicrobial [13].

*Staphylococcus aureus* and some *Streptococcus* species are common cases of septic olecranon bursitis (Table 2). Other reported organisms are shown in Table 2, which is patterned after Reilly and Kamineni [50]. Notably, their table contains multiple errors, which we have corrected (see footnote to Table 2). Septic olecranon bursitis is usually a bacterial infection caused by skin lesions or by secondary spread from initial cellulitis into a pre-traumatized superficial bursa [87]. Factors that increase the risk of acquired septic olecranon bursitis are persistent pressure on the bursa due to work demands, corticosteroid therapy, rheumatoid arthritis, lupus, gout, and other conditions that reduce immunity. Alcoholics have a higher incidence of septic olecranon bursitis due to their higher risk of trauma and immunosuppression [39,50,88]. Similarly, smoking increases the chances of olecranon bursitis and generally slows wound healing [89,90]. Eradication of olecranon bursitis can take three times longer in immunocompromised patients [39,91].

Our patient had prior surgery for an infection in the same location, which caused a subcutaneous adhesion at the upper aspect of the olecranon bursa region (Figure 1). In addition to his alcoholism and current smoking habit, we suggest that this adhesion and additional scar tissue along the incision of the prior debridement surgery resulted in reduced perfusion, potentially impairing immunity and impeding adequate antibiotic tissue levels. Asseray et al. [80] report an increased likelihood of infection in cases with prior surgery in the same area. In a comprehensive review of septic bursitis in anatomical locations, Zimmerman et al. [44] provide an illustrative case and also state that a previous septic olecranon bursitis can increase the likelihood of developing a subsequent septic olecranon bursitis.

## Conclusions

Although *C. acnes* has a well-known propensity for false-positive cultures as typically reported in post-operative shoulder infections, treatment for our patient’s septic olecranon bursitis/osteomyelitis was successful only after several surgical debridements and a prolonged course of IV and oral antibiotics that targeted it as the presumptive causal organism. However, it was still possible that *C. acnes* was a contaminant/superinfection, and another organism was the culprit, such as a *Streptococcus* or *Mycobacterium* species, which was eradicated by the treatment regime targeted for *C. acnes*. The infection was successfully eradicated with IV antibiotics and an extended course of oral antibiotics. We speculated that this extended course of oral antibiotics following IV antibiotics reflected the ability of *C. acnes* or another anaerobic bacteria species to form a biofilm, our patient’s immunocompromised state and impaired healing capacity resulting from alcoholism and smoking, his prior case of septic bursitis leaving subcutaneous scar tissue, and bone involvement. This case illustrates the often challenging empirical management of a musculoskeletal infection when the only organism isolated is *C. acnes*, which might be a contaminant but successful eradication requires continued treatment as if it is the causal organism.
